# Minipuberty assessment in newborns with hypoxic ischemic encephalopathy treated with therapeutic hypothermia: a single-center case–control study

**DOI:** 10.3389/fped.2023.1201668

**Published:** 2023-06-21

**Authors:** Lucia Lanciotti, Rossella Sica, Laura Penta, Francesca Parisi, Alberto Argentiero, Maurizio Radicioni, Giuseppe Di Cara, Francesca Di Genova, Alberto Verrotti, Stefania Troiani, Susanna Maria Roberta Esposito

**Affiliations:** ^1^ Department of Pediatrics, University of Perugia, Perugia, Italy; ^2^ Pediatric Clinic, Department of Medicine and Surgery, Azienda Ospedaliera-Universitaria, University of Parma, Parma, Italy; ^3^ Department of Neonatal Intensive Care Unit and Neonatal Pathology, S. Maria Della Misericordia Hospital of Perugia, Piazzale Giorgio Menghini 1, Perugia, Italy

**Keywords:** minipuberty, therapeutic hypothermia, hypoxic ischemic encephalopathy, hypothalamic–pituitary–gonadal axis, hypothermia

## Abstract

**Introduction:**

The aim of our single-center case–control study is to evaluate whether minipuberty occurs in patients with hypoxic ischemic encephalopathy (HIE) who underwent therapeutic hypothermia (TH). We intend to conduct this evaluation by confronting the values of luteinizing hormone (LH) and follicle-stimulating hormone (FSH) and the values of testosterone in males and estradiol in females between newborns with HIE and in subsequent TH and healthy controls.

**Methods:**

We enrolled 40 patients (age: 56–179 days; 23 males), of whom 20 met the inclusion criteria for the case group and who underwent TH. A blood sample was taken from each patient at approximately 10 weeks of age to evaluate FSH and LH from the serum samples of all patients and to evaluate 17-beta estradiol (E2) and testosterone levels, respectively, from the serum samples of female and male patients.

**Results:**

It was found that minipuberty occurred in the case group patients, with no significant differences reported from the control group and with hormonal serum levels comparable to healthy infants of the control group (FSH 4.14 mUI/ml ± 5.81 SD vs. 3.45 mUI/ml ± 3.48 SD; LH 1.41 mUI/ml ±1.29 SD vs. 2.04 mUI/ml ±1.76 SD; testosterone in males 0.79 ng/ml ± 0.43 SD vs. 0.56 ng/ml ± 0.43 SD; 17-beta estradiol in females 28.90 pg/ml ± 16.71 SD vs. 23.66 pg/ml ± 21.29 SD).

**Discussion:**

The results of the present study may pave the way for further research and the evaluation of more possible advantages of TH.

## 1. Introduction

Neonatal hypoxic ischemic encephalopathy (HIE) is an important cause of neonatal death and disability, occurring in 1.5 per 1,000 livebirths (1). Therapeutic hypothermia (TH) has been proved to reduce cerebral injury and improve the neurological outcome secondary to HIE in newborns at or near term with evolving moderate-to-severe HIE (2, 3). Since TH is not a “magic bullet,” the need to research and evaluate the efficacy of add-on therapies that can further improve outcomes after HIE is becoming increasingly evident (4–6).

The hypothalamic–pituitary–gonadal (HPG) axis activates for the second time after fetal life in the neonatal period, mainly in the first 3–6 months of life, and this process is called “minipuberty.” The absence of placental steroids suppressing the HPG axis after birth causes a progressive reduction of negative feedback, allowing gonadotropin levels to rise approximately between days 6 and 10 after birth (7–9). Sex differences in the luteinizing hormone (LH) and follicle-stimulating hormone (FSH) peak and decline have been reported (10). Serum LH levels, in fact, are dominant in males, while serum FSH levels are dominant in females with a higher peak and a longer lasting period until 3–4 years of age (10–13). In male neonates, the increasing serum levels of LH stimulate testosterone secretion from the Leydig cells, reaching a peak between 1 and 3 months of age and declining to prepubertal values by 6–9 months of age (10, 14). Similarly, the FSH peak stimulates inhibin B and Anti-Mullerian Hormone (AMH) production from the Sertoli cells and the proliferation of the Sertoli and germ cells. AMH peaks at 3 months; it declines and remains stable throughout childhood until puberty, at which point there is a progressive decline to adult levels (9, 15). Estradiol levels are high at birth in the cord blood of both sexes and gradually decrease in the first days of life. A new rise in these levels can be seen in girls after the first week of life, with high and fluctuating levels until at least the 6th month of life (16), reflecting the FSH trend and the cycles of maturation and atrophy of the ovarian follicles. AMH levels rise at 3 months of age (17), probably because of the postnatal proliferation of granulosa cells and the parallel development of the ovarian follicles.

Data from the literature reveal that minipuberty seems to allow the development of the genital organs, especially in preterm infants (18), and create the basis for future fertility. Our aim in this study is to evaluate whether minipuberty occurs in patients with HIE who underwent TH by confronting the values of LH and FSH and the values of testosterone in males and estradiol in females in this population and in healthy controls.

## 2. Material and methods

### 2.1. Study design and population

This was a single-center case–control study. We enrolled 40 patients (age: 56–179 days; 23 males). Four of the forty patients were preterm infants (median gestational age 39.3 weeks).

The inclusion criteria for the case group were as follows: age range between 60 and 180 days, evidence of peripartum asphyxia, eligibility for TH [an Apgar score of 5 or less at 10 min; mechanical ventilation or resuscitation at 10 min; cord pH < 7.1, or an arterial pH < 7.1 or a base deficit of 12 or more within 60 min of birth; evidence of encephalopathy according to Sarnat staging (19); abnormalities in video-electroencephalography recordings], and evidence of having undergone TH. The inclusion criteria for the control group were age range between 60 and 180 days, no evidence of complications during childbirth, and an Apgar score ≥9 at 1 and 5 min.

The exclusion criteria for the study were major congenital abnormalities recognizable at birth (metabolic and/or chromosomal abnormalities, malformations and congenital infection) and present and previous evidence of availing any therapies.

Twenty of the enrolled patients met the inclusion criteria for the case group (age: 64–128 days; males: 11; preterm: 1), while the remaining 20 met the criteria for the control group and were, therefore, enrolled as controls (age: 56–179 days; males: 12; preterm: 3).

The study protocol was approved by the Ethics Committee of Umbria Region CEAS N. 3115/17.

Written informed consent was obtained from the parents of the study patients.

### 2.2. Methods

Asphyxiated infants admitted at our third-level Neonatal Intensive Care Unit were treated with whole-body cooling to an esophageal temperature of 33.5°C maintained for 72 h by using the servo-controlled Blanketrol III hyper-hypothermia system (Cincinnati Sub-Zero, OH, United States). Eligibility test and treatment of the asphyxiated infants were performed according to the established criteria and methods (20).

Video-electroencephalography recordings were performed at admission, as an entry criterion, during the rewarming phase, and in response to clinical seizures. The esophageal probe (lower third of the esophagus) was checked with a chest x-ray to determine whether its placement was in the correct position and subsequently verified whether any abnormal swings of the esophageal probe occurred or any blanket temperatures were noted. All infants received phenobarbital and fentanyl infusion for sedation and pain control as specified by institutional protocol (respectively, 10 mg as the initial loading dose and then 5 mg/kg, based on the blood concentration of the drug on the fourth day, and 0.5–2 µg/kg/h). Esophageal, skin, rectal, and blanket temperatures, vital signs, intravenous fluids, urine output, and inotropic support were recorded on an hourly basis on a predesigned data form. The type and number of transfused blood components were also documented. Laboratory assessments for determining platelet count, prothrombin time, activated partial thromboplastin time, fibrinogen levels, and D-dimer levels were performed on admission and every 24 h during the same period.

A blood sample was taken from each patient between the second and the third month of life (approximately 10 weeks of chronological age) to evaluate the hormonal status in the serum of minipuberty. We analyzed FSH and LH from the serum samples of all patients and 17-beta estradiol (E2) and testosterone, respectively, from the serum samples of female and male patients. The samples were simultaneously quantified using a chemiluminescent immunoassay (UniCel DxI 800, Access Beckman Coulter ®, Immunoassay System).

### 2.3. Statistical analysis

For making a descriptive analysis of the continuous variables, mean and standard deviation (SD) were calculated. E2 values were considered a dichotomous variable deriving from the threshold of 20 applied to the quantitative value, and frequency was reported. Fisher’s exact test was used to find whether there were any differences between the case and the control groups. The non-parametric Wilcoxon rank-sum test was performed to compare the mean for the continuous variables between the groups. *P*-values of less than 0.05 were considered statistically significant. Statistical analyses were performed by using STATA Statistical Software (Release 11 College Station, TX).

## 3. Results

Among the 40 enrolled infants, 20 were part of the case group and 20 belonged to the control group.

**Figure 1:**
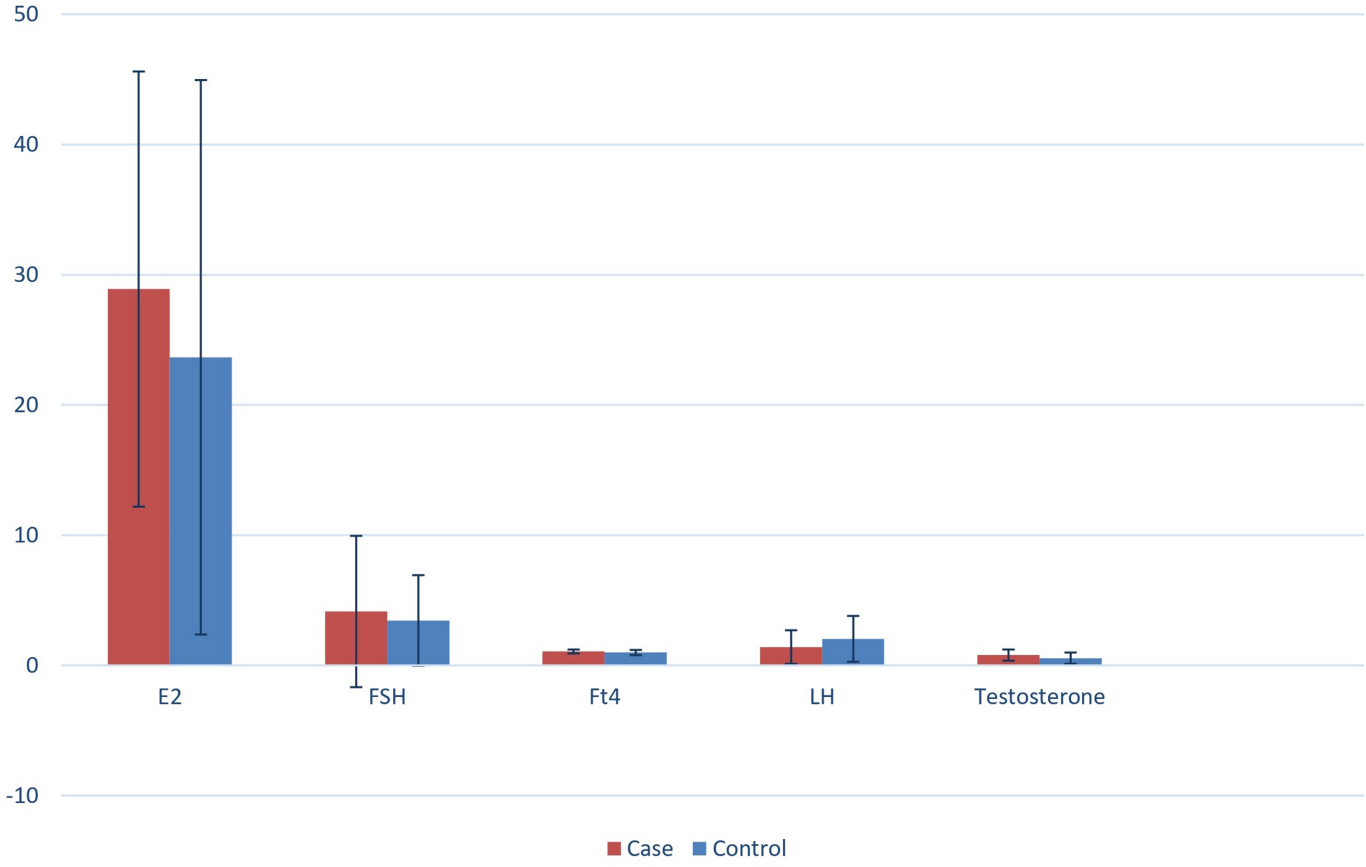
Serum samples concentrations of FSH, LH, testosterone and E2 of both the case and the control group.

In Figure 1, the serum sample concentrations of FSH, LH, testosterone, and E2 of both the case and the control groups are shown. In Table 1, the serum levels of FSH and LH are shown, and these were comparable as no statistically significant difference between the two groups was detected (FSH 4.14 mUI/ml ± 5.81 SD vs. 3.45 mUI/ml ± 3.48 SD and LH 1.41 mUI/ml ±1.29 SD vs. 2.04 mUI/ml ±1.76 SD, respectively). Also, differences in the age of the patients were not statistically significant (89.15 ± 15.23 days vs. 92.55 ± 31.56 days).

**Table 1:** Serum levels of FSH, LH , testosterone and E2 are comparable as no statistically significant difference among the case and the control group was detected.

Variable	Cases (*n* = 20)	Controls (*n* = 20)	*p*-value
Age (days)	89.15 ± 15.23	92.55 ± 31.56	NS
E2 (pg/ml)	28.90 ± 16.71	23.66 ± 21.29	NS
E2 < 20 (pg/ml)	4 (20%)	5 (25%)	NS
FSH (uUI/ml)	4.14 ± 5.81	3.45 ± 3.48	NS
LH (uUI/ml)	1.41 ± 1.29	2.04 ± 1.76	NS
Testosterone (ng/ml)	0.79 ± 0.43	0.56 ± 0.43	NS

E2, estradiol; LH, Luteinizing hormone; FSH, Follicle-stimulating hormone.

In both the study groups, mean serum LH levels were found to be higher in males than in females (levels in females of the case group: 0.65 mUI/ml; levels in females of the control group: 0.39 mUI/ml vs. levels in males of the case group: 2.02 mUI/ml; levels in males of the control group: 2.99 mUI/ml). However, FSH levels were higher in females than in males (levels in females of the case group: 7.49 mUI/ml; levels in females of the control group: 6.65 mUI/ml vs. levels in males of the case group: 1.39 mUI/ml; levels in males of the control group: 1.31 mUI/ml).

The mean serum levels of testosterone in males (0.79 ng/ml ± 0.43 SD in the case group vs. 0.56 ng/ml ± 0.43 SD in the control group) and the mean serum levels of E2 in females (28.90 pg/ml ± 16.71 SD in the case group vs. 23.66 pg/ml ± 21.29 SD in the control group) in both groups were comparable, with no statistically significant differences existing between the groups (Table 1).

Serum E2 levels were found to be <20 pg/ml in 20% of female patients of the case group (*n* = 4) and in 25% of female patients of the control group (*n* = 5). These data had no statistically significant value (Table 1).

## 4. Discussion

The main aim of our single-center case–control study was to evaluate whether minipuberty occurred in newborns with HIE who underwent TH. The method of evaluation was by analyzing the differences between the values of LH and FSH and between the values of testosterone in males and estradiol in females in patients with HIE who underwent TH and in healthy controls.

The results of our study demonstrated that the manner of occurrence of minipuberty was similar in both the case and the control groups. In fact, there was no statistical significance between the mean serum levels of FSH, LH, E2, and testosterone in both groups.

As reported in the results section of the study, in both groups, mean serum LH levels were higher in males than in females, while FSH levels were higher in females than in males, thus confirming the findings from the literature (11, 21). Moreover, in our male population, testosterone secretion levels were also high, reflecting the increasing number of Leydig cells in the testicular tissue under LH stimulation until the third month. In female patients of both groups, E2 mean serum concentration levels were similar to those reported in the literature. In fact, a study by Schmidt et al. reported that median serum E2 levels in girls at 3 months of age were approximately 30.0 pmol/L (range < 18–100) (15). However, it is known that individual E2 levels in females may show considerable fluctuation in the first months of life, which is mainly attributed to the cycles of maturation and atrophy of the ovarian follicles (21).

As with preterm patients, in our study, prematurity did not seem to affect the timing of the onset of the surge in postnatal gonadotropin levels. In fact, the gonadotropin levels began to increase at the same time after birth as in full-term patients, thus confirming the findings from the literature. In particular, in our population, there were four preterm infants (females: 3; male: 1), and we found medium serum levels of LH and FSH of 0.85 and 4.3075 mUI/ml, respectively. Moreover, in our study, the LH and FSH serum levels appeared to be similar to those in the full-term newborns.

Data from the literature revealed that the surge in FSH and LH levels was higher and prolonged in preterm infants than in full-term infants, especially in females (21–23). Kuiri-Hänninen et al. compared full-term and preterm males by measuring urinary gonadotropin levels and testosterone levels in serial urine samples and comparing the results with testicular and penile growth. The trends in LH and testosterone secretion levels were found to be significantly higher in preterm than in full-term infants (12). A study by Greaves et al., which analyzed LH and FSH levels in a population of 82 premature infants born under a 30-week gestation, reported that prematurity was related to significantly high gonadotropin levels in girls (LH levels from 0.1 to 13.4 IU/L and FSH levels from 0.3 to 4.6 IU/L in male preterms vs. LH levels from 0.2 to 54.4 IU/L and FSH levels from 1.2 to 167.0 IU/L in female preterms) (23). These hormonal patterns may reflect an immaturity of the negative feedback system in the HPG axis, and the highest levels of FSH in preterm females may be attributed to immature ovaries that do not seem to be capable of producing estrogen of sufficient quantity that will inhibit the secretion of gonadotropin.

The limitations of our study are its small population sample size, the lack of previous studies in the literature, and the fact that for ethical reasons it is not possible to have newborns with HIE who did not undergo TH as controls.

The strengths of our study are that this is the first case–control study to analyze hormonal functions and minipuberty in infants with HIE treated with TH. Minipuberty occurred in the case group with hormonal serum levels comparable to healthy infants and with no statistically significant differences reported from the control group. In this study, we discovered that minipuberty itself seemed to play an important role as a marker of a functional HPG axis in infants with HIE treated with TH. Nevertheless, we suggest that TH may have a role in preventing brain damage and preserving subcortical functions.

We expect that the results of this study may pave the way for further research to evaluate more possible advantages of TH.

## Data Availability

The original contributions presented in the study are included in the article, further inquiries can be directed to the corresponding author.
